# Blood transfusion in severe dengue infection: a case series

**DOI:** 10.1186/s13256-022-03716-w

**Published:** 2023-01-18

**Authors:** S. A. M. Kularatne, Chamara Dalugama, Madhara Rajapakse, Sithara Warnasooriya, Manoji Pathirage, Udaya Ralapanawa, Thilak Jayalath

**Affiliations:** Department of Medicine, University of Peradeniya, Kandy, Sri Lanka

**Keywords:** Bleeding, Blood, Complications, Dengue, Liver, Management

## Abstract

**Background:**

Dengue is still a recurrent challenge to the global population, without specific antiviral therapy. Clinical management strategies are aimed to mitigate the deaths. The use of blood products in dengue is recommended mainly in cases of bleeding.

**Case presentation:**

We prospectively collected data on Sri Lankan dengue cases in the Teaching Hospital, Peradeniya, Sri Lanka from 2017, and selected ten severe cases where blood transfusions were involved in the management. The series comprises seven females and three males, with a median age of 36 years (range 12–53 years). All patients were critically ill at the time of blood transfusion, with dramatic stabilization of vital parameters after the transfusions. Only one patient had detectable bleeding, while five patients had occult blood loss as indicated by dropping hematocrit. Even though four patients had stable hematocrit, they had metabolic acidosis. Two patients had a very high increase of hepatic transaminases along with acidosis. Two patients had myocarditis with dropping hematocrit, suggestive of occult bleeding.

**Conclusions:**

Clinical deterioration despite fluid management commonly occurs due to occult bleeding in dengue infection. Blood transfusion is lifesaving in such cases of blood loss, acidosis, and severe hepatic damage. The mechanism of this effect needs an explanation, such as enhanced oxygen delivery to the tissues and hemostasis to hypothesize a few possibilities.

## Background

Dengue is an arboviral infection endemic to the Asia–Pacific region, with diverse clinical manifestations ranging from asymptomatic disease to dengue hemorrhagic fever (DHF) and multiple organ failure [1]. Uncomplicated dengue infection is no different to other viral fevers in which recovery happens over a few days. But dengue hemorrhagic fever, if not diagnosed and managed in time, can be fatal. Four dengue viral serotypes are known to cause illness, where cross immunity may predispose to severe infection [2] Sri Lanka is one example where dengue infection is rampant over last two decades, leading to significant mortality [3]. The Ministry of Health of Sri Lanka has a dengue task force and introduced dengue management guidelines 11 years ago, which are updated regularly [4]. However, the number of deaths still remains high, with more than 100 deaths in 2019, a reduction compared with 400 deaths in 2017 [9, 10]

The mainstay of management for dengue hemorrhagic fever is meticulous monitoring of vital parameters, urine output, and hematocrit (Hct), and titrating fluids to match the plasma leakage in the critical phase [4]. The guideline on the management of dengue in adults mainly discusses fluid management and states limited indications for blood transfusion [4]. However, in clinical practice, prompt utilization of blood transfusion seems to be lifesaving in severe dengue infection based on clinical judgment. However, the use of blood transfusion in dengue management is neither studied in detail nor widely practiced, except in overt bleeding. We believe that appropriate use of blood transfusion will certainly reduce the deaths from dengue. With this intention, the following ten authentic cases of severe dengue infection are presented from the Teaching Hospital, Peradeniya, Sri Lanka, where blood transfusion appeared to be a lifesaving treatment. The objectives of the study are to evaluate the severity of the dengue infection using a scoring system, to elucidate the imminent indication for blood transfusion, and to show the rapidity of improvement of patient’s well-being after transfusions.

## Methods

We prospectively collected data of dengue cases in the Teaching Hospital, Peradeniya, Sri Lanka from 2017 to 2019, and selected ten severe cases where blood transfusions were involved in the management. The clinical and laboratory information was recorded from the bed head tickets. All the patients received standards of care according to the national guidelines, and indication for the blood transfusion was recorded for each patient. A severity score comprising 20 points within eight major criteria of clinical parameters was introduced by the authors, which were applied prior to and after the blood transfusions.

## Results

### Case 1 (dropping hematocrit in a DHF patient with clinical deterioration)

A 34-year-old Sri Lankan female was admitted with fever with myalgia for 3 days duration with a positive nonstructural protein 1 (NS1) antigen test for dengue. On day 4 of the fever, the patient complained of postural giddiness and vomiting. She had a pulse rate of 100 beats/minute with a supine blood pressure of 100/80 mmHg. On day 4, her white blood cell count was 2.65 × 10^6^/L, with a platelet count of 66 × 10^3^/L, aspartate transaminase (AST) of 109 U/L, and alanine transaminase (ALT) of 42 U/L. Serum albumin was 29 g/L with very low non-fasting cholesterol (2.44 mmol/L). Bedside ultrasonography confirmed the presence of free fluid in the hepato–renal pouch with marked gallbladder wall edema. The patient was receiving intravenous and oral fluids. The hematocrit (Hct) increase was noted from 37% to 41% and the patient was managed as critical phase of dengue hemorrhagic fever. During the 22nd hour of the tentative critical phase, a drop of Hct from 40 to 34 was noted without an apparent clinical improvement. During this hour, the patient had a rise in pulse rate from 68 beats/minute to 94 beats/minute, with a blood pressure of 88/65 mmHg. The decision to transfuse blood was taken and the patient was transfused 400 mL of whole blood from the 23rd to the 24th hour of the critical phase. Following blood transfusion, Hct improved to 38%, with an increase in blood pressure to 110/70 mmHg and pulse rate stabilized around 84 beats/minute. The rest of the critical phase was uncomplicated. The patient was discharged on day 7. The patient had no obvious blood loss in the form of melena, hematemesis, menorrhagia, or hematuria. Also, there were no cutaneous bleeding manifestations.

### Case 2 (dropping Hct in a DHF patient)

A 46-year-old Sri Lankan female presented to the Teaching Hospital, Peradeniya (THP) with a 3-day history of fever with arthralgia and myalgia. Her NS1 antigen was positive, and on admission her platelet count was 135 × 10^3^/L with a white blood cell count of 3.2 × 10^6^/L. On admission, her Hct was 38% with a hemoglobin level of 12.4 g/dL. On day 4 of the illness, the patient was complaining of postural symptoms and abdominal pain. Reduction of urine output was noted over the last 6 hours, but blood pressure and heart rate were within normal range. In-ward Hct was 31% (a 7% reduction from the baseline). Bedside ultrasonography revealed free fluid in the hepato–renal pouch with gallbladder wall edema. Her venous lactate level was 4.5 mmol/L. Diagnosis of dengue hemorrhagic fever with bleeding was made, although clinically overt bleeding was not identified. The patient received 250 mm of whole blood followed by 150 mL the next hour and 100 mL per hour for 2 hours until the Hct was restored to 40%. Following the blood transfusions, the patient experienced a feeling of well-being followed by an increase in urine output. Venous lactate level reduced to 2.4 mmol/L 1 hour after transfusion. The rest of the critical phase was uncomplicated and the patient made a full recovery and was discharged on day 6.

### Case 3 (clinically deteriorating patient with worsening acidosis with stable Hct)

A 40-year-old Sri Lankan male presented with an acute febrile illness with arthralgia and myalgia for 3-days duration. His dengue NS1 antigen was positive. On day 6 of illness, he was found to have free fluid in the hepato–renal pouch and small pleural effusion on ultrasound examination. His hemodynamics and urine output was maintained well within normal limits. He was monitored as critical phase of dengue hemorrhagic fever. Reduction of urine output was noticed during the 22nd–24th hours of the critical phase, with deterioration of the general condition with postural symptoms. No rise in Hct was noted but his heart rate increased to 102 beats/minute from 80 beats/minute, with narrowing pulse pressure to 20 mmHg. Venous blood gas revealed metabolic acidosis with a bicarbonate of 12 mmol/L, lactate level of 3.9 mmol/L, and serum corrected calcium of 1.78 mmol/L. Acidosis was corrected with 8.4% sodium bicarbonate and hypocalcemia was corrected with 10% calcium gluconate. His Hct remained stable during this period without dropping. The decision was taken to transfuse blood despite no rise in Hct, which could not account for clinical deterioration. A volume of 400 mL of whole blood was transfused over 2 hours. Immediately following the blood transfusion, hemodynamic parameters stabilized and urine output increased. Venous blood gas done after blood transfusion showed improvement in bicarbonate (24 mmol/L). The rest of the critical phase was unremarkable. The patient was discharged on day 8.

### Case 4 (rising transaminases with static Hct in a DHF patient)

A 24-year-old Sri Lankan female presented with a history of fever and myalgia of 4-days duration. On admission, her hemoglobin was 10.2 g/dL with a white blood cell count of 2.56 × 10^6^/L and a platelet count of 86 × 10^3^/L. Her blood tests revealed iron deficiency anemia with possible thalassemia trait. She was found to have a plasma leak on day 5 of illness, and critical phase monitoring was started. Although she had ultrasonic fluid leakage, her pulse rate was 68 beats/minute at the onset, with a blood pressure of 110/60 mmHg and a Hct of 29%, which is compatible with low hemoglobin. She maintained good urine output. On the 20th hour of the critical phase, she had a pulse rate of 88 beats/minute with normal blood pressure. Over the next few hours, she complained of abdominal pain, headache, and postural symptoms. Her transaminases were on the rise, with ALT 300 U/L (from the baseline value of 95 U/L on admission) and AST 326 U/L, with a venous lactate level of 3.1 mmol/L. It was decided to transfuse whole blood to increase the Hct as there was no expected rise in the Hct despite plasma leaking, and liver enzymes were increasing. The patient was given 450 mL of whole blood and Hct increased to 33% and apparent improvement in clinical well-being was noticed, along with a reduction in pulse rate to 56 beats/minute, good urine output, and normalization of venous lactate level. Her subsequent transaminases decreased and the patient was discharged on day 8.

### Case 5 (DF with gastrointestinal blood loss and severe anemia)

A 12-year-old Sri Lankan girl was admitted to the THP with a history of fever of 5-days duration. On admission, she had melena with an episode of hematemesis. She complained of hematuria of 1-day duration. She was pale and ill-looking. Her vital parameters were stable with a pulse rate of 100 beats/minute and blood pressure of 120/70 mmHg. Bedside scan did not reveal any evidence of plasma leakage. NS1 and dengue immunoglobulin (Ig)M were both positive, with negative IgG, and her serotype was identified as DEN 2. She was managed as having primary dengue fever with bleeding manifestations. Her liver enzymes were only mildly elevated (AST 87 U/L and ALT 56 U/L) and her clotting profile was normal. Full blood count revealed hemoglobin of 7 g/dL and platelet count of 17 × 10^9^/μL. She was transfused with 500 mL of blood and four units of platelets. She did not enter into the critical phase and was discharged on day 8 with a rising platelet count with stable hemoglobin.

### Case 6 (DHF with shock needing massive transfusion)

A 14-year-old Sri Lankan male was managed for dengue fever. On day 5 of the illness, the patient developed postural symptoms, vomiting, and profuse diarrhoea. On examination, he was tachycardic with a pulse rate of 130 beats/minute and blood pressure of 90/60 mmHg. Bedside ultrasound scan showed evidence of plasma leak to pleural and peritoneal cavities. He was diagnosed as having DHF complicated with septic shock and gastroenteritis. Broad-spectrum intravenous antibiotics (ceftriaxone and metronidazole) were started to cover the sepsis after taking blood and urine cultures. His C-reactive protein was 114 mg/L. During critical phase monitoring, the patient suddenly deteriorated with a sudden drop in blood pressure needing multiple fluid boluses including normal saline and dextran. A drop in Hct was noted from 36 to 30 during initial decompensation. Although there was no clinically overt bleeding, the patient needed continuous transfusion of whole blood amounting to 9 pints over 20 hours to maintain blood pressure and urine output. In addition to fluid resuscitation, the patient needed an intravenous noradrenaline infusion to maintain blood pressure. The patient was gradually weaned off vasopressors and Hct was stabilized, and he was discharged on day 8 after admission.

### Case 7 (DHF with very high transaminases suggestive of liver necrosis with stable Hct)

A 53-year-old Sri Lankan male was admitted with an acute febrile illness of 4 days, with arthralgia and retro-orbital pain. In addition, he had marked right upper abdominal pain with vomiting. His NS1 antigen was positive and he was managed as dengue hemorrhagic fever. On admission, the patient had a pulse rate of 100 beats/minute and supine blood pressure of 120/80 mmHg and standing blood pressure of 110/90 mmHg. The patient had severe liver involvement on admission, with aspartate transaminase (AST) of 27,220 U/L and alanine transaminase (ALT) of 11,100 U/L. Serum bilirubin was elevated, with a direct fraction of 45%. The prothrombin time (PT) was 17.1 seconds (control 12 seconds) and activated partial thromboplastin time (APTT) was 142.7 seconds (control 29 seconds). The venous blood gas showed a compensated metabolic acidosis with bicarbonate of 15 mmol/L with a lactate level of 5.8 mmol/L. The patient was started on standard DHF management and in addition, intravenous N-acetyl cysteine infusion was started. An antibiotic (third generation cephalosporin) was given. His Hct remained static at around 36–38%. The management team decided to transfuse whole blood at a rate of 100 mL/minute to reach and maintain Hct of 45% for 8 hours, which resulted in a dramatic reduction of venous lactate from 5.8 to 3.2 mmol/L, with a gradual reduction in transaminases. The patient was discharged on day 7 of admission, with near-normal transaminases without any long-term sequel.

### Case 8 (DHF with high transaminases–liver necrosis with acute kidney injury with stable Hct)

A 40-year-old Sri Lankan female presented with acute febrile illness with myalgia and arthralgia, and developed plasma leakage on day 4 of the illness. She had DHF complicated with fulminant liver necrosis with alanine transaminase (ALT) of 6542 U/L, aspartate transaminase (AST) of 30,617 U/L, and deranged clotting with elevated serum lactate. She developed acute kidney injury needing continuous renal replacement therapy. She was managed with intravenous N-acetyl cysteine. Although there was no overt bleeding or dropping Hct, the decision was taken to transfuse whole blood, aiming for a higher Hct to increase oxygenation to deliver at the tissue level. She had an uneventful recovery and was discharged on day 9 after admission.

### Case 9 (DHF with myocarditis and drop of Hct)

A 16-year-old Sri Lankan girl presented with a febrile illness with positive serology and NS1 antigen. She developed postural symptoms on day 4 of the illness, and her pulse rate was 120 beats/minute, blood pressure of 100/80 mmHg, with < 0.5 mL/kg urine output for 6 hours. Bedside ultrasound scan revealed free fluid in the hepato–renal pouch. Initial Hct was 38%. Her platelet count was 45,000/mcL at the time of diagnosis of plasma leak. Following the resuscitation with crystalloids, her urine output improved and Hct stabilized around 35%. At hour 24 of the critical phase, the patient was tachycardic with a pulse rate of 110 beats/minute and tachypneic with respiratory rate of 24 breaths/minute. She had nasal flaring and complained of difficulty in breathing and had an oxygen saturation of 93% on air needing supplementary oxygen. On auscultation, lung fields were clear. A 12-lead electrocardiogram (ECG) revealed widespread T wave inversions and a bedside echocardiogram revealed mild global hypokinesia with an ejection fraction (EF) of 47% suggestive of myocarditis. Her Hct at 24 hours was 32%. There was no clinically overt bleeding. The venous blood gas showed elevated lactate of 3.3 mmol/L with a normal bicarbonate level. A decision was made to transfuse blood at a lower rate to increase Hct to 36–38% without overloading. Following the blood transfusion, the patient showed clinical improvement, with improvement in urine output and a reduction in pulse rate to 88 beats/minute. Venous lactate level 1 hour after the blood transfusion was 2.8 mmol/L. The rest of the critical phase was uneventful. On discharge she had normal ECG tracing and follow-up 2D echo revealed good Left Ventricular (LV) function with EF > 60%

### Case 10 (DHF with myocarditis plus possible pulmonary hemorrhages causing Hct drop)

A 36-year-old previously health Sri Lankan female presented with a history of acute febrile illness of 3-days duration with positive NS1 antigen. On day 5 of illness, she complained of shortness of breath. She had marked bradycardia with a pulse rate of 48 beats/minute. Bedside 2D echo showed good LV function with no regional wall motion abnormalities. On day 7 of the illness, she was having postural symptoms with reduced urine output. Bedside ultrasonography revealed mild bilateral pleural effusions with free fluid in the hepato–renal pouch. She had marked desaturation with dropping Hct, reduced urine output, and rising lactate. At the 23rd hour of the critical phase, she was transfused 400 mL of packed cells of blood over 3 hours, which resulted in improvement in urine output and in general well-being. Her chest X-ray revealed diffuse alveolar opacifications. She was given a 50 mL/hour packed cell transfusion for 6 hours to maintain her hematocrit. She needed high-flow oxygen with a bag to maintain saturation above 90%. Gradually her lactate level improved. Her oxygen was weaned off over the next 48 hours and she was discharged on day 11 without sequel.

### Summary of 10 cases

This series comprises seven females and three males, with a median age of 36 years (range 12–53 years). The summarized clinical data are presented in Table 1. We introduced a severity score to give a numerical figure for the clinical severity of dengue infection (Table 2). This score comprises 20 points within eight major criteria of clinical parameters. Table 3 presents the application of this score to the index cases before and after blood transfusion, and the dramatic drop of severity scores after blood transfusion denoting the lifesaving therapeutic effect of blood transfusion.

**Table 1:** Summary of the clinical status of patients before blood transfusion

No.	Patient details	Fever day of blood transfusion	Clinical and laboratory parameters at the time of blood transfusion										Immediate indication for the blood transfusion	Time taken to improve the clinical status
			BP	PR	plt	Hct	WBC	SpO_2_	Overt bleeding	ALT	AST	Lactate		
1	34F	4	88/65	94	23	34	2.6	98	No	42	109		Dropping Hct in a DHF patient with clinical deterioration	4 hours
2	46F	4	100/60	88	42	31	1.2	98	No	64	52	4.5	Dropping Hct in a DHF patient	1 hour
3	40M	6	90/70	102	36	38	1.8	98	No	65	45	3.9	Deteriorating patient with worsening acidosis with stable Hct	2 hours
4	24F	5	110/60	88	22	33	2.6	98	No	300	326	3.1	Rising transaminases with static Hct + acidosis	12 hours
5	12F	5	120/80	80	17	21	1.6	96	Yes	56	87		DF with gastrointestinal blood loss, severe anaemia	4 hours
6	14M	5	90/60	130	12	30	1.4	97	Yes	112	134		DHF with diarrhea, shock, and dropping Hct and acidosis	4 hours
7	53M	5	110/70	100	12	37	2.3	98	No	11,100	27,220	5.8	DHF with massive liver necrosis with stable Hct + acidosis	10 hours
8	40F	4	100/70	88	28	35	1.1	99	No	6542	30,617	14.5	DHF with massive liver necrosis with acute kidney injury and stable Hct + acidosis	24 hours
9	16F	4	100/80	120	26	35	2.6	93	No	84	42	3.1	DHF with myocarditis and low Hct	12 hours
10	36F	7	120/70	54	52	31	1.6	85	Yes	64	49		DHF with myocarditis + pulmonary hemorrhages + hypoxaemia, acidosis, low Hct	12–24 hours

Patient details (age/gender); BP, blood pressure (mmHG); PR, pulse rate; Plt, platelet count (× 10^6/^/L); Hct, hematocrit (%); WBC, white cell count (× 10^9^/L); AST (IU/L); ALT (IU/L); lactate (mmol/L); SpO_2_, peripheral oxygen saturation (%); DHF, dengue hemorrhagic fever; NO, number; F, Female; M, Male; BP, Blood pressure; PR, Pulse rate; Plt, Platelets; HCT, haematocrit; WBC, White cell count; SpO_2_. Oxygen saturation; ALT, Alanine aminotransaminase; AST, Aspartate aminotransaminase; DHF, Dengue Haemorrhagic Fever; DF, Dengue Fever

**Table 2:** Clinical severity score of dengue infection

1. General condition	
a. Ill look, malaise, headache	Score 1
b. Postural dizziness, cold peripheries, venous collapse	Score 1
2. Hematological	
a. Bleeding (GI, GU, gum bleeding, skin bleeding)	Score 1
b. Drop of PCV by 5 without clinical	Score 1
c. Platelet count < 50,000/mcL	Score 1
3. Cardiac	
a. Heart rate > 100	Score 1
b. Systolic blood pressure < 90 mmHg	Score 1
c. ECG: diffuse T inversions/ST changes	Score 1
d. Low ejection fraction in echo	Score 1
4. Pulmonary	
a. Tachypnea RR > 20 cycles/minute	Score 1
b. SpO_2_ < 95%	Score 1
5. Renal	
a. Urine output < 0.5 mL/kg for > 6 hours	Score 1
b. Rising serum creatinine > 1.5× baseline	Score 1
6. Liver	
a. Rising serum bilirubin ×3 times	Score 1
b. Transaminases(AST and ALT) > 1000	Score 1
c. INR > 1.5 or APTT > 1.5 times the control	Score 1
7. Nervous	
a. Drowsy/GCS < 14/15	Score 1
b. EEG changes	Score 1
8. Venous blood gas parameters	
a. Lactate > 3 mmol/L	Score 1
b. Venous bicarbonate < 15 mmol/L	Score 1
Total score 20	

GI, Gastrointestinal; GU, Genitourinary; PCV, Packed cell volume; GCS, Glasgow Coma Scale; RR Respiratory rate

**Table 3:** Severity score before and after the blood transfusion

	Patient 1	Post transfusion	Patient 2	Post transfusion	Patient 3	Post transfusion	Patient 4	Post transfusion	Patient 5	Post transfusion	Patient 6	Post transfusion	Patient 7	Post transfusion	Patient 8	Post transfusion	Patient 9	Post transfusion	Patient 10	Post transfusion
1. General condition																				
a. Ill look, malaise, headache	1	0	1	0	1	0	1	0	1	0	1	0	1	0	1	0	1	0	1	0
b. Postural dizziness, cold peripheries, venous collapse	1	0	1	0	1	0	1	0	1	0	1	0	1	0	1	0	1	0	1	0
2. Hematological																				
a. Bleeding (GI, GU, gum bleeding, skin bleeding)	0	0	0	0	0	0	0	0	1	0	0	0	0	0	0	0	0	0	1	0
b. Drop of PCV by 5 without clinical improvement	1	0	1	0	0	0	0	0	1	0	1	0	0	0	0	0	0	0	0	0
c. Platelet count < 50,000/mcL	1	1	0	0	1	1	1	0	1	1	1	1	1	0	0	0	1	1	0	0
3. Cardiac																				
a. Heart rate > 100 < 50	1	0	0	0	1	0	0	0	1	0	1	0	1	0	1	0	1	0	1	0
b. Systolic blood pressure < 90	1	0	0	0	1	0	0	0	0	0	1	0	0	0	1	0	0	0	0	0
c. ECG: diffuse T inversions/ST changes	0	0	0	0	0	0	0	0	0	0	0	0	0	0	0	0	1	0	1	0
d. Low ejection fraction on echo	0	0	0	0	0	0	0	0	0	0	0	0	0	0	0	0	1	0	0	0
4. Pulmonary																				
a. Tachypnea RR > 20 cycles/minute	1	0	0	0	1	0	0	0	0	0	1	0	1	0	1	0	1	0	1	1
b. SpO_2_ < 95%	0	0	0	0	0	0	0	0	0	0	0	0	0	0	1	0	1	0	1	1
5. Renal																				
a. Urine output < 0.5 mL/kg for > 6 hours	1	0	1	0	1	0	0	0	0	0	1	0	1	0	1	0	1	0	1	0
b. Rising serum creatinine > 1.5× baseline	0	0	0	0	0	0	0	0	0	0	0	0	0	0	1	0	0	0	0	0
6. Liver																				
a. Rising serum bilirubin ×3 times	0	0	0	0	0	0	0	0	0	0	0	0	1	0	1	1	0	0	0	0
b. Transaminases(AST and ALT) > 1000	0	0	0	0	0	0	0	0	0	0	0	0	1	0	1	1	0	0	0	0
c. INR > 1.5 or APTT > 1.5 times the control	0	0	0	0	0	0	0	0	0	0	0	0	1	0	1	0	0	0	0	0
7. Nervous																				
a. Drowsy/GCS < 14/15	0	0	0	0	0	0	0	0	0	0	0	0	1	0	1	0	0	0	0	0
b. EEG changes	N/A	N/A	N/A	N/A	N/A	N/A	N/A	N/A	N/A	N/A	N/A	N/A	0	0	0	0	N/A	N/A	N/A	N/A
8. Venous blood gas parameters																				
a. Lactate > 3 mmol/L	1	0	1	0	1	0	1	0	0	0	1	0	1	0	1	0	1	0	1	0
b. Venous bicarbonate < 15 mmol/L	1	0	0	0	1	0	0	0	0	0	1	0	1	0	1	0	0	0	1	0
Overall score	10	1	5	0	9	1	4	0	6	1	10	1	12	0	14	2	10	1	10	2

GI, Gastrointestinal; GU, Genitourinary; PCV, Packed cell volume; GCS, Glasgow Coma Scale; RR, Respiratory rate

## Discussion

Our case series describes ten confirmed cases of dengue infection with a range of clinical manifestations that deteriorated despite standard management, but made rapid improvement with a blood transfusion.

The use of red cells and other blood products in the management of dengue fever is highly debated and controversial due to the lack of evidence-based guidelines. According to the national guidelines for management of dengue fever in adults in Sri Lanka [4], blood transfusions are indicated in severe bleeding that can be recognized by detecting bleeding visually, a significant drop of hematocrit without clinical improvement, or persistent or worsening metabolic acidosis in patients. This is in agreement with World Health Organization (WHO) guidelines [5]. Indian national guidelines recommend red cell transfusions for patients with overt blood loss of 10% or more of blood volume, and in refractory shock with declining hematocrit [6]. Although there is evolving evidence on the use of red cells in indications other than the above guideline-based recommendations, there is still a lack of randomized trials and high-quality evidence to guide the use [7, 8].

However, we observed blood transfusions to be lifesaving in many deteriorating dengue patients outside the stipulated indications. In clinical practice, the decision to give a blood transfusion is often taken after a long delay, leading to irreversible consequences. Therefore, this decision should be taken promptly and without delay. We observed that all our patients had dramatic improvement in their clinical status after blood transfusion. If not for blood transfusion, these patients would have further deteriorated irreparably. As randomized controlled clinical trials are not feasible, a case series of this nature helps clinicians to make a decision on blood transfusions in cases of severe dengue.

In this case series, only case 5, a 12-year-old girl, had visible bleeding. She had significant bleeding into the gastrointestinal tract along with severe thrombocytopenia without derangement of the liver profile or plasma leak. This is a very rare situation of primary dengue causing bleeding and thrombocytopenia. Bleeding manifestations commonly occur in both DF and DHF as a result of many causes. More than thrombocytopenia, platelet dysfunction causing impairment in platelet aggregation results in bleeding [11]. The life span of platelets is reduced in DF and the presence of antibodies against platelets has been described [10]. However, platelet production in the bone marrow is also reduced [13]. Clotting derangements have also been described in DF in some studies without liver involvement [14, 15]. Patients with severe liver involvement can have severe coagulopathy. Prolonged hypotension can lead to gut ischemia resulting in major gastrointestinal hemorrhages in severe cases of DHF [16]

The most common type of bleeding in dengue is occult blood loss into undetectable sites inside the body. This could be either focal or diffuse, manifesting as clinical deterioration. In DHF, even though plasma leak is the hallmark, a risk of occult bleeding always exists. Clinical vigilance and analyzing surrogate markers can alert clinicians to occult bleeding. A reduction in Hct out of proportion to intravenous fluid resuscitation is the standard measure to detect significant occult hemorrhages. In our series, five patients (cases 1, 2, 6, 9, and 10) had falling Hct along with clinical deterioration out of proportion to plasma leak and hypotension. At the same time, they had other associated life-threatening manifestations such as acidosis, hypoxemia, and cardiac dysfunction. However, rapid improvement was observed with blood transfusions. Therefore, meticulous monitoring of Hct is important in DHF and measured judgment is needed in giving the blood transfusion to suit the clinical condition of each patient. Where occult bleeding can occur is an unanswered question. In autopsy studies, macroscopic bleeding was detected in the lungs, liver, and peritoneal cavity [17], but further studies are needed to ascertain bleeding into tissue plains such as muscles, which can accommodate large amounts of occult loss from seeping vasculature.

In this series, four patients did not have a reduction in Hct, but they still improved with blood transfusion. Of these, three patients had stable Hct, while case 4 had an increase in Hct together with clinical deterioration. What was common among these patients was metabolic acidosis. In this scenario, what was the mechanism by which blood transfusion improved the deteriorating clinical parameters? We can assume that the transfused blood would have increased the oxygen-carrying capacity of blood to the tissues, thereby correcting the acidosis [18, 19]. Another mechanism could be an expansion of circulatory volume and a reduction in the rate of plasma leak by increasing the oncotic pressure of the plasma. Therefore, it is important to detect acidosis due to circulatory failure. This could be achieved by monitoring all vital parameters and urine output. In DHF, hemoconcentration due to selective plasma leakage clinically manifests in the form of tachycardia, narrowing of pulse pressure, and oliguria [20]. However, in the absence of a change in hematocrit, if clinical parameters deteriorate, it could be assumed that plasma leakage is occurring in the presence of occult bleeding, which warrants transfusions and meticulous titration of intravenous fluids.

Two patients had severe liver involvement and benefited from blood transfusions. Hepatic dysfunction in dengue can vary from asymptomatic elevation of transaminases to fulminant liver necrosis. Mild-to-moderate increases in transaminases are common in DF and DHF. Elevated transaminases in 74.2% of patients with serologically confirmed dengue illness were described by Souza *et al*. [21]. Dalugama *et al*. described two cases of severe liver involvement in DHF [18, 19]. Different mechanisms have been postulated to explain the hepatic dysfunction, including the hepatotropism of the dengue virus, immune mechanisms, and liver ischemia [22]. The patient will have intravascular volume depletion due to plasma leakage, for which the body will operate autoregulatory mechanisms through which perfusion to vital organs such as the heart and brain will be maintained at the expense of “non-vital” organs such as skin and gut. Developmentally, the liver is a gut derivative and can suffer the same ischemia as the gut. Severe liver involvement can manifest as very high transaminases, deranged clotting, and increasing serum lactate level. Red cell transfusions in this context will increase the Hct and the oxygen carrying capacity of the blood, and will mitigate the ischemic damage to the liver [18, 19]. In cases 7 and 8, blood transfusions improved liver injury by a dramatic reduction in the venous lactate levels and gradual reduction in the transaminases.

Two patients had myocarditis, and their deterioration was out of proportion to the cardiac dysfunction. Myocarditis and cardiac dysfunction are recognized complications of dengue illness [23, 24]. Patients can develop dengue myocarditis, which might manifest in the form of breathlessness, palpitations, extreme bradycardia or tachycardia, or more commonly clinical deterioration without a rise in Hct (that is, plasma leak). An electrocardiogram might show ST changes. Overloading with crystalloids in this scenario to improve blood pressure or urine output would be counterproductive. When myocarditis is associated with occult bleeding, as in cases 9 and 10, the only surrogate marker of bleeding is Hct, which may show a drop. However, the decision to give a blood transfusion is difficult, particularly the volume and the rate of transfusion.

The use of red cells is not without risks. Patients with dengue fever are at risk of fluid overload and pulmonary edema for many reasons which include reabsorption of fluid in DHF during the latter part of the critical phase and convalescence, underlying myocardial dysfunction, overenthusiastic use of fluids during management. So the use of red cells might put the patients at increased risk of volume overload. Use of blood products can be associated with febrile nonhemolytic transfusion reactions, allergic reactions, alloimmunization, and transfusion-associated infections [25–27]. An interesting study in Brazil concluded that the use of blood components without criteria exposes patients to further risks. The study recommended that adherence to WHO guidelines for the use of blood components reduces costs and hospitalization times for patients hospitalized with dengue. But generalizing this study to make inferences has limitations as the majority of patients received platelet transfusion and pack cells were received by less than 10% of the study population [28]. Therefore, there is a fine balance between the risks and benefits of the use of blood products. The decision to use red cells in the management of a dengue patient must be based on individual assessment of the patient and used only if the benefits outweigh the risks. The ten cases presented here are such examples of using blood products for a favorable clinical outcome, in parallel with standard management according to the guidelines.

## Conclusion

We report ten cases of severely ill dengue patients, where the indication for blood transfusion was promptly recognized during the clinical course and to the patients’ benefit. As dengue does not have a specific antiviral treatment, clinical management is governed by analyzing the escalation of problems during the course of the illness, where meticulous use of blood transfusion could be lifesaving in highly selected categories of patients.

## Data Availability

Not applicable.

## Abbreviations

Hct: Hematocrit
DF: Dengue fever
DHF: Dengue hemorrhagic fever
AST: Aspartate transaminase
ALT: Alanine transaminases
IgM: Immunoglobulin M
APPT: Activated partial thromboplastin time
PT: Prothrombin time
